# Probiotic Potential of Yeasts Isolated from Fermented Beverages: Assessment of Antagonistic Strategies Against *Salmonella enterica* Serovar Enteritidis

**DOI:** 10.3390/jof10120878

**Published:** 2024-12-17

**Authors:** Silvia Cristina Vergara Alvarez, María Dolores Pendón, Ana Agustina Bengoa, María José Leiva Alaniz, Yolanda Paola Maturano, Graciela Liliana Garrote

**Affiliations:** 1 Instituto de Biotecnología, Universidad Nacional de San Juan, Av. San Martín 1109 (O), San Juan 5400, Argentina; scristina.vergara@gmail.com (S.C.V.A.); majoleiva4@gmail.com (M.J.L.A.); paolamaturano@gmail.com (Y.P.M.); 2 Consejo Nacional de Investigaciones Científicas y Técnicas (CONICET), Godoy Cruz 2290, Ciudad Autónoma de Buenos Aires 1425, Argentina; mdolorespendon@gmail.com (M.D.P.); bengoaagustina@gmail.com (A.A.B.); 3 Centro de Investigación y Desarrollo en Criotecnología de Alimentos (CIDCA), CONICET-UNLP-CIC, Street 47 and 116, La Plata 1900, Argentina

**Keywords:** kefir, wine, probiotic, non-*Saccharomyces* yeasts, antagonistic activity, *Salmonella*

## Abstract

Global concern about pathogenic resistance to antibiotics is prompting interest in probiotics as a strategy to prevent or inhibit infections. Fermented beverages are promising sources of probiotic yeasts. This study aimed to evaluate the antagonistic effects of *Kluyveromyces marxianus*, *Wickerhamomyces anomalus*, and *Pichia manshurica* strains from kefir and wine against *Salmonella enterica* serovar Enteritidis in intestinal epithelial cells. The ability of these yeasts to adhere to Caco-2/TC-7 cells was evaluated, as well as their influence on the ability of *Salmonella* to associate and invade these cells. The behavior of the pathogen was analyzed by (a) incubation of enterocytes with yeast before adding *Salmonella*, (b) co-incubation of *Salmonella* with yeast before contact with the enterocytes, and (c) incubation of *Salmonella* with yeast metabolites before contact with enterocytes. All yeast strains demonstrated adherence to Caco-2/TC-7 cells (33–100%) and effectively inhibited *Salmonella* invasion. Among the treatments, co-culture showed the greatest effect, reducing *Salmonella* association and invasion by more than 50%. Additionally, these yeasts modulated the epithelial immune response, significantly decreasing CCL20-driven luminescence by 60–81% (*p* < 0.0001). These results highlight the potential of yeasts from fermented beverages as probiotics to counteract *Salmonella* infections, offering a promising alternative in the fight against antibiotic resistance.

## 1. Introduction

The resistance of pathogens, including *Salmonella* species, to common antibiotics is currently a serious threat to global health, resulting in significant morbidity and mortality worldwide [1]. The overuse and inadequate management of antibiotics have led to the emergence and spread of resistant and multidrug-resistant pathogens in both nosocomial and community settings [2]. Furthermore, over the years antibacterial treatments have become less effective due to the impact of antibiotics on normal intestinal microbiota [3].

Microorganisms living in complex microbial communities often resort to the production of antimicrobial compounds that, by diverse mechanisms, kill and/or inhibit the growth of competing microorganisms [4]. Likewise, competition for nutrients and adhesion sites in the host mucosa, or the induction of proinflammatory responses for the elimination of pathogens, have been observed in vitro and in vivo [5,6]. The need to reduce antibiotic resistance has directed research towards the characterization of the antimicrobial properties of non-pathogenic bacteria and fungi [7], with probiotics being an attractive strategy.

It is accepted that probiotics are live microorganisms that can be beneficial to the consumer when administered in adequate amounts [8]. Helmy et al. proposed that probiotics beneficially affect the host using different mechanisms, such as altering the microbial community associated with the organism, ensuring enhanced utilization of substrates, improving its immune system, or upgrading the host reaction toward certain sicknesses, among other aspects [9]. Probiotics must survive the conditions of the upper gastrointestinal tract and then persist in the intestine to provide beneficial effects to the host. To stay for a certain period of time, microorganisms possess several mechanisms to adhere to the intestinal epithelial cells [10].

Most probiotics available on the market belong to the lactic acid bacteria group and the genus *Bifidobacterium*. Other relevant bacterial species commonly used as probiotics are *Bacillus* spp., *Enterococcus* spp., *Escherichia coli*, and *Weissella* spp. [11]. There is constant interest in the search for new strains of probiotic microorganisms and, within them, yeasts constitute an attractive alternative that have not been extensively explored yet [12,13]. One of the main advantages of probiotic yeasts over probiotic bacteria is that yeasts are not affected by antibacterial drugs usually administered to treat enteropathogenic infections and, thus, they can be used as complementary therapy. Unlike bacteria, yeasts do not spread antibiotic resistance genes, and their translocation has never been reported [14]. Furthermore, yeasts have the ability to reduce the adherence of pathogens to mucosal surfaces and have been suggested to play a positive role in diseases caused by a change in the healthy gut microbiota, such as inflammatory bowel diseases (IBDs) [15].

In particular, the adhesion of probiotic yeasts to the intestinal epithelium can elicit antagonism to enteropathogenic microorganisms both directly and indirectly. Direct antagonism takes place by competitive exclusion for binding sites or nutrients, by steric hindrance, and by temporary colonization, where bioactive compounds produced by the microorganisms such as organic acids, ethanol, killer toxins, biosurfactants, and bioactive peptides display an antimicrobial effect. Indirect antagonism may occur by modulating the immune system through the induction of pro- and/or anti-inflammatory signaling pathways [16]. Furthermore, probiotics can trigger antimicrobial effects by co-aggregation with pathogenic microorganisms, preventing the growth and biofilm formation of the pathogens. Auto-aggregation, hydrophobicity, and biofilm production by probiotics are also considered mechanisms that favor cellbarrier formation that could inhibit the colonization of pathogens [17,18]. Adherence to epithelial cells is a desirable probiotic trait, as it facilitates colonization of the human gut, preventing elimination of probiotics by peristalsis and providing a competitive antagonism against pathogens [19,20]. Several mechanisms allow the adhesion of microorganisms to intestinal epithelial cells, including interaction through pili, fimbriae, surface proteins, secretion systems, or mucosal layers, among others. In the case of yeasts, auto-aggregation has been associated with the promotion of colonization in the human intestine, immunomodulation of the colonic mucosa, and prevention of pathogenic infections [21]. The adhesive property is also attributed to certain cell surface proteins (known as adhesins) present on the surface of yeast cells that bind to carbohydrate residues, such as in the mucins of epithelial cells or certain amino acids on the surface of other cells [22].Yeasts have been also described to be able to rapidly adapt their adhesion properties to new environments due to the phenotypic variability and plasticity detected in closely related strains [22].

As is well known, fermentative environments are highly selective, which would indicate that microorganisms isolated from fermented products would adapt to competitive and stressful conditions such as those of the gastrointestinal tract. The probiotic potential of yeast strains isolated from different fermented beverages such as kefir, water kefir, and oenological environments has been previously evaluated for their ability to withstand low pH, bile salts, and some of them have been selected as probiotic candidates [12,23,24,25]. This study aimed to investigate the potential of these yeasts to antagonize *Salmonella* sp. within the intestinal environment. Specifically, their adhesion to Caco-2/TC-7 intestinal epithelial cells, their impact on the association and invasion of *Salmonella enterica* serovar Enteritidis (*S. Enteritidis*) to enterocytes, and their ability to modulate the epithelial innate immune response were evaluated using the Caco-2:luc CCL20 reporter system, as an indicator of anti-inflammatory potential.

## 2. Materials and Methods

### 2.1. Yeasts and Bacterial Strains and Maintenance

*Kluyveromyces marxianus* CIDCA 8154 and CIDCA 9121 were isolated from kefir and sugar kefir, respectively [23], and belong to the Collection of Centro de Investigación y Desarrollo en Criotecnología de Alimentos (CIDCA, La Plata, Argentina), (Table 1). *K. marxianus* CIDCA 8154 (LPSc 1427) and CIDCA 9121 (LPSc 1426) have been deposited in the Spegazzini Collection of La Plata (LPSc, La Plata, Argentina), a public access culture collection belonging to the WDCM (WDCM1001). *Wickerhamomyces anomalus* PB97, PB98, PB99, and *Pichia manshurica* PB54 were isolated from environments associated with winemaking, grape musts throughout different stages of spontaneous fermentations during the 2004 and 2011 harvests (Cuyo region, Argentina), belonging to the collection of autochthonous microorganism strains of the Institute of Biotechnology, Faculty of Engineering, National University of San Juan. *Wickerhamomyces anomalus* PB97 (COMIM 4428), PB98 (COMIM 4429), PB99 (COMIN 4430), and *P. manshurica* PB54 (COMIM 4431) have been deposited in the Collection of Microorganisms INTA Mendoza (CoMIM), located at the National Institute of Agricultural Technology (INTA).

Strains were identified by conventional morphological, physiological, and biochemical assays according to Kurtzman et al., and a molecular assay as described by Esteve-Zarzoso et al. [26,27]. Frozen stock cultures were stored at −80 °C in YPD medium (yeast extract 10 g/L, bacterial peptone 20 g/L and glucose 20 g/L) with 50% (*v*/*v*) glycerol. Kefir yeasts were cultured in whey permeate obtained from the dairy industry in powder form resuspended in distilled water at 100 g/L, while the oenological origin yeasts were activated in YPD at pH = 4.6. All strains were incubated at 30 °C for 24 h, without shaking (kefir strains), and with shaking at 100× *g* (oenological strains).

*Salmonella enterica* serovar Enteritidis (*S. Enteritidis*) CIDCA 101 isolated from a human clinical sample from the Hospital de Pediatría Prof. Juan P. Garrahan (Buenos Aires, Argentina) was grown in nutrient broth (Biokar Diagnostics, Beauvais, France) for 18 h at 37 °C and stock.

### 2.2. Adhesion of Yeasts to Caco-2/TC-7 Cells

Caco-2/TC-7 cells that model the mature enterocytes of the large intestine were routinely grown following the procedure described by Zavala et al. [28]. To evaluate the adhesion capacity of selected yeasts, 24-well plates of Caco-2/TC-7 cells grown in a confluent monolayer (for 7 days) were used. Activated yeasts were washed (4200× *g* for 5 min) twice with phosphate-buffered saline (PBS; in *w*/*v*: 0.9% NaCl, 0.08% Na_2_HPO_4_, 0.014% KH_2_PO_4_, pH 7.2) and resuspended in Dulbecco’s Modified Eagle’s Minimal Essential Medium (DMEM, GIBCO BRL Life Technologies, Rockville, MD, USA) adjusted to an optical density (OD) of approximately 0.5 at 610 nm. Under sterile conditions, each monolayer was washed twice with PBS at room temperature and 0.5 mL of microbial suspension was added to each well. Plates were incubated at 37 °C for 1 h in a controlled atmosphere (5% CO_2_–95% air). Subsequently, monolayers were washed 3 times with PBS and lysed with 0.5 mL of sterile milli-Q water; the plate was incubated again for 1 h at 37 °C under a controlled atmosphere. To determine the number of viable yeasts adhered to Caco-2/TC-7, the entire volume was taken and appropriate dilutions in physiological solution were plate in duplicates on YGC agar (yeast extract 5 g/L, glucose 20 g/L, chloramphenicol 0.1 g/L, agar 14.9 g/L) by drop method. Colony counts were performed after 24 h of incubation at 30 °C. All experiments were performed in triplicate. The results were expressed as percentage of yeast adhesion:

(1) $$\mathrm{ADH}\%=\left(\frac{\mathrm{CFUf}}{\mathrm{CFUi}}\right)\times 100$$

where CFUf represents the final colony-forming units of adherent yeasts and CFUi represents the initial colony-forming units of yeast added to the well.

### 2.3. Auto- and Co-Aggregation Assays

The aggregation capacity of yeasts was investigated according to Prabhurajeshwar and Chandrakanth with minor modifications [29]. Cultures of each yeast strain (24 h at 30 °C) and *S. Enteritidis* (16 h at 37 °C) were centrifuged (4200× *g* for 5 min), washed twice, and resuspended in PBS. The yeasts (10^7^ CFU/mL) and the pathogen (10^7^ CFU/mL) were subjected to auto-aggregation tests in monoculture. To test their ability to co-aggregate, 10^7^ CFU/mL of yeast with 10^7^ CFU/mL of the pathogen were mixed. Two milliliters of yeast, bacteria, or mixed culture suspension were added to aglass cuvette, and OD at 610 nm was measured in a spectrophotometer (Metrolab 330, Buenos Aires, Argentina) every 10 min without disturbance for 60 min. The auto-aggregation coefficient was calculated at 1 h according to:

(2) $$\mathrm{AC}\%=\left(\frac{\mathrm{ODt}-\mathrm{ODi}}{\mathrm{ODt}}\right)\times 100$$

where ODi represents the initial optical density and ODt is the optical density at time 60 min.

### 2.4. Effect of Yeasts on the Association and Invasion of Salmonella Enteritidis CIDCA 101 into Caco-2/TC-7 Cells

Association and invasion of *S. Enteritidis* to Caco-2/TC-7 cells was evaluated according to Zavala et al. with modifications [28]. First, Caco-2/TC-7 obtained from the American Type Culture Collection in confluent monolayers were washed twice with PBS. Subsequently, 0.5 mL of *S. Enteritidis* suspension in DMEM (10^7^ CFU/mL) was added and incubated 1 h at 37 °C in a controlled atmosphere (5% CO_2_–95% air). For association assays, monolayers were washed three times with PBS after incubation and then lysed adding 0.5 mL of sterile milli-Q water. The number of viable *S. Enteritidis* associated (adhering plus invading) with Caco-2/TC-7 cells was determined by plating the appropriate dilutions on nutrient agar and counting colonies after incubation (24 h at 37 °C). The percentage of *S. Enteritidis* association was calculated as:

(3) $$\mathrm{SC}\%=\left(\frac{\mathrm{CFUasc}}{\mathrm{CFUi}}\right)\times 100$$

where CFUasc is the final count of associated *S. Enteritidis* and CFUi corresponds to the initial count added to the well.

*Salmonella* invasion was determined by counting bacteria inside Caco-2/TC-7 cells. For this purpose, the monolayers incubated with *S. Enteritidis* (as previously described) were treated with 0.5 mL/well of gentamicin (100 μg/mL PBS) for 1 h at 37 °C. Finally, cells were lysed, and colonies were counted as described above. The percentage of internalized *S. Enteritidis* was calculated as:

(4) $$\mathrm{INV}\%=\left(\frac{\mathrm{CFUint}}{\mathrm{CFUi}}\right)\times 100$$

where CFUint represents the final count of internalized *Salmonella* while CFUi is the initial *Salmonella* count added to the well.

In order to evaluate the effect of yeasts on *S. Enteritidis* association/invasion of intestinal epithelial cells, the behavior of the pathogen was analyzed in three different treatments:

(a) Effect on the enterocytes pretreated with yeast (yeast effect, YE), where cells were pre-incubated with 0.5 mL of yeast suspension in DMEM (OD_610nm_ = 0.5) for 1 h at 37 °C in 5% CO_2_–95% air and then washed three times with PBS before adding *Salmonella* suspension. (b) Effect of co-incubation of *Salmonella* with the yeast prior to contact the enterocytes (CC), where yeast (10^7^ CFU/mL) and *Salmonella* (10^7^ CFU/mL) suspensions were mixed and co-incubated in PBS (pH 7.2) for 1 h at 37 °C. Then, the mixed suspension was centrifuged (2900× *g* for 4 min), resuspended in the same volume of DMEM and 0.5 mL of the mixture was added to each well. (c) Effect of the metabolites produced by the yeast (cell-free supernatant, CFS) on *Salmonella* prior to contact with epithelial cells. In this case, CFS was obtained by centrifugation (4200× *g* for 5 min) and filtration (0.22 µm pore diameter) of a fresh yeast culture. *Salmonella* (10^7^ CFU/mL) was incubated 1 h at 37 °C in yeasts’ CFS and, afterwards, the suspension was centrifuged, the pellet was resuspended in DMEM, and 0.5 mL of this suspension was added to each well. *S. Enteritidis* association and invasion after these three different treatments was determined as described above.

### 2.5. Immunomodulation on Caco-2-CCL20: Luc Cells

For in vitro characterization of the yeasts immunomodulatory properties, the Caco-2-ccl20: luc reporter system provided by Dr M. Rumbo group was employed. Briefly, human colonic epithelial cell line Caco-2 stably transfected with a luciferase reporter construction under control of the CCL20 promoter (Caco-2-CCL20: luc) were maintained and routinely grown according to Iraporda et al. [30,31].

Wet biomass of each yeast strain was suspended in DMEM (GIBCO BRL Life Technologies Rockville, Rockville, MD, USA) at OD_600nm_ = 0.5 (~10^7^ CFU/mL). Cultured Caco-2 CCL20: luc cells were incubated with the yeast suspension for 30 min using serum-free medium in 48-well plates. Cells were then stimulated with *Salmonella tyhimurium* flagellin (FliC, 1 μg/mL) and incubated for 5 h at 37 °C in a 5% CO_2_–95% air atmosphere. A basal condition without any treatment was included in all experiments, whereas FliC was used as a control for 100% induction of pro-inflammatory response. Then, cells were lysed with Lysis Buffer (Promega, Madison, WI, USA). Labsystems Luminoskan TL Plus luminometer (Thermo Scientific, Waltham, MA, USA) was used to measure luciferase activity using a Luciferase Assay System (Promega, Madison WI, USA). Luminescence was normalized to the stimulated control cells and expressed as the percentage of normalized average luminescence ± standard deviation (NAL ± SD) of at least three independent experiments.

### 2.6. Statistical Analysis

Comparisons between more than two treatments were analyzed by one-way analysis of variance (ANOVA), to determine differences between groups using Dunnett’s post-test to compare each group to a control group (association and invasion assays) or Tukey’s test to compare means between groups (in assays of adhesion and immunomodulation). Analyses were performed with GraphPad version 8.02 (San Diego, CA, USA). *p* values less than 0.05 were considered statistically significant.

## 3. Results

### 3.1. Yeasts Adhesion to Caco-2/TC-7 Cells, and Auto- and Co-Aggregation Capacity

As observed in Table 1, the adhesion capacity of yeasts to Caco-2/TC-7 cells varies significantly both at the species and origin matrix levels, with the lowest values recorded by *K. marxianus* CIDCA 8154 (33.89%), and the highest by *W. anomalus* PB 97 (100%). Regarding the aggregation capacity after 1 h, the oenological yeasts showed very low auto-aggregation values that could be considered negligible. On the other hand, yeasts isolated from kefir showed significantly higher values, reaching percentages of auto-aggregation greater than 88% (Table 1 and Supplementary Figure S1). It is interesting to note that under the conditions tested, no co-aggregation was observed between *S. Enteritidis* and any of the yeasts tested (Supplementary Figure S2).

### 3.2. Effect of Potential Probiotic Yeast Strains on the Association and/or Invasion of S. Enteritidis into Caco-2/TC-7 Cells

**Figure 1:**
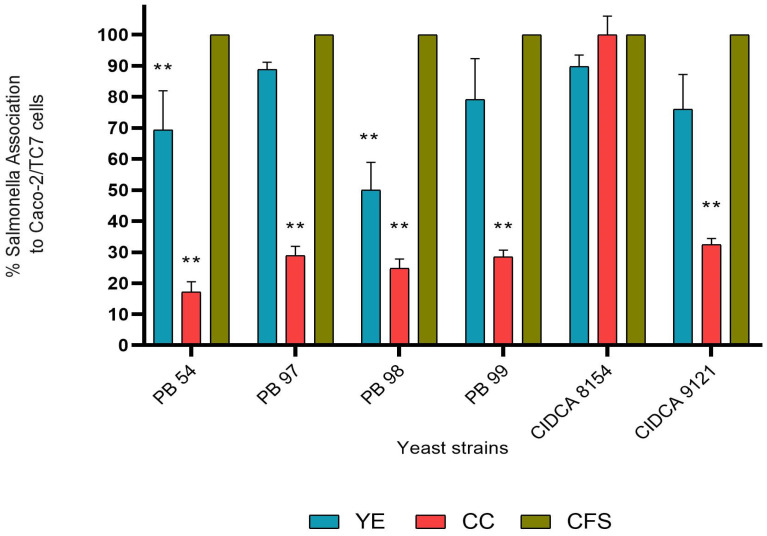
Effect of yeasts strains on the association of *S. Enteritidis* to Caco-2/TC-7. Percentage of association of *S. Enteritidis* to Caco-2/TC-7 cells treated with yeasts before pathogen addition (YE), *S. Enteritidis* pre-cultured with yeasts (CC), and *S. Enteritidis* pretreated with yeasts cell-free culture supernatants (CFS). ** statistically significant differences (*p* ≤ 0.05) compared to the control representing 100% association.

One of the first steps in *Salmonella* sp. infection is tissue colonization, for which its association with epithelial cells is crucial. If this interaction could be interfered with in any way, the activity of the pathogen would be inhibited. The results indicate that when the enterocytes are previously treated with the yeasts *W. anomalus* PB 98 and *P. manshurica* PB 54, the association of *S. Enteritidis* to epithelial cells is significantly reduced by 50 and 30%, respectively, probably through the so-called barrier effect (Figure 1). On the other hand, except for *K. marxianus* CIDCA 8154, all yeasts significantly decreased the percentage association of *S. Enteritidis* between 67 to 82% by pretreatment of the pathogen with yeast strains in co-culture (CC). Additionally, no effect was observed on the association of *S. Enteritidis* to Caco-2/TC-7 cells when pretreated with any of the yeasts cell-free culture supernatants (CFS).

**Figure 2:**
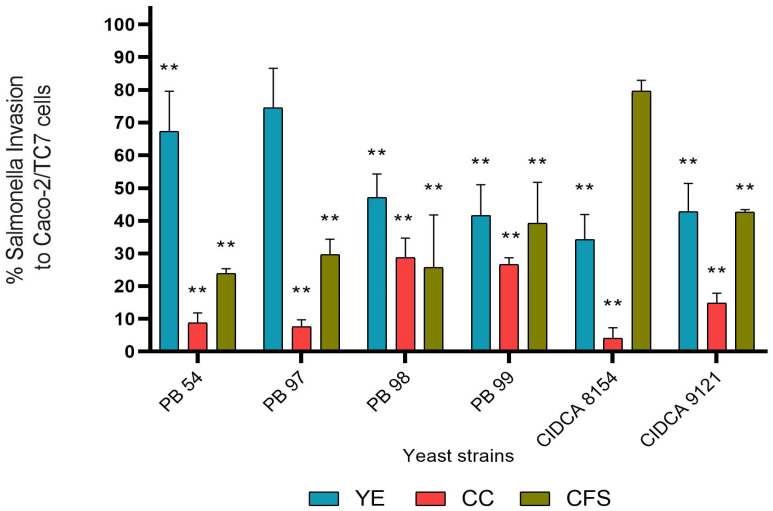
Effect of yeast strains on the invasion of *S. Enteritidis* to Caco-2/TC-7. Percentage of invasion of *S. Enteritidis* to Caco-2/TC-7 cells treated with yeasts before pathogen addition (YE), *S. Enteritidis* pre-cultured with yeasts (CC), and *S. Enteritidis* pretreatment with yeast cell-free culture supernatants (CFS). ** statistically significant differences (*p* ≤ 0.05) compared to the control representing 100% invasion.

Regarding the percentage of *S. Enteritidis* invasion ofCaco-2/TC-7 cells, treatment with all yeast strains, except PB 97, significantly reduced it between 32 and 65% (Figure 2). For the co-culture treatment, all strains showed a reduction in the percentage of *S. Enteritidis* invasion greater than 70%, being *K. marxianus* CIDCA 8154 the most effective leading to a decrease of 95.95%. When the pathogen was incubated with the CFS of yeasts, there was a significant reduction in its invasion capacity of between 57 and 76%, except in the case of the CFS of CIDCA 8154 strain, which only reduced it by 20%, with respect to the control.

In general, the treatment with the most notable effect on most strains was the CC pre-incubation since it decreased the percentage of association and invasion of *S. Enteritidis* by more than 50% in most strains; only *K. marxianus* CIDCA 8154 had no effect on the association but was notably the most effective in reducing the invasion percentage.

### 3.3. Immunomodulation of Epithelial Innate Response by the Yeasts

**Figure 3:**
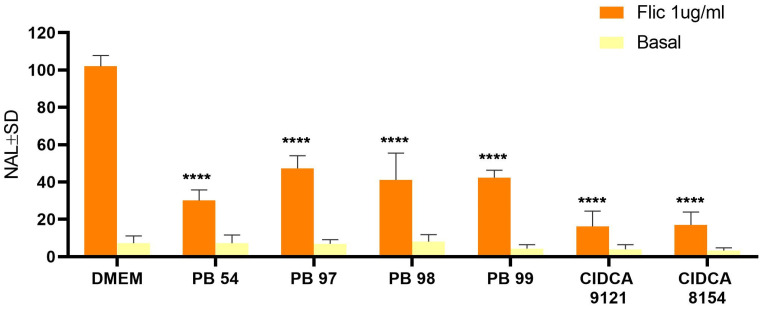
Effect of yeasts strains on the immunomodulation of Caco-2-CCL20: luc cells. Reporter cells were stimulated with flagellin (1 µg/mL) after pretreatment with yeasts. Luciferase activity was determined in a cell lysate 5 h after stimulation. Results representative from two different experiments are shown. Results are expressed as normalized luciferase activity (NAL), using the levels of stimulated cells in the absence of yeast cells as 100% of activation. **** *p* < 0.0001.

Since probiotic features may depend on the strain and their physiological status, the beneficial properties of yeasts, such as their capacity to modulate the innate immune response, should be demonstrated. Thus, yeasts from different environments were analyzed regarding their ability to modulate the response to pro-inflammatory stimuli on intestinal epithelial cells. For this purpose, Caco-2 genetically modified (Caco-2 ccl20: luc) were used, which contain the CCL20 promoter-controlled luciferase gene and, in turn, responds to stimulation with flagellin. Therefore, there is a direct correlation between increased luciferase activity and increased inflammatory response, as described by Nempont et al. [30]. All tested yeasts displayed a strong capacity to reduce Caco-2 innate immune response induced by FliC, lowering the luminescence in different values between 60 and 81% and demonstrating the anti-inflammatory capacity of strains (Figure 3).

## 4. Discussion

In the context of the global increase in antibiotic resistance, our findings highlight the importance of exploring alternatives such as probiotic yeasts to combat enteric infections. This study focused on assessing the impact of specific yeast strains isolated from fermented products, such as kefir and winemaking environments, on the pathogenic capacity of *Salmonella enterica* serovar Enteritidis.

The yeasts evaluated in this study have previously demonstrated biological safety, tolerance to gastrointestinal conditions, and various beneficial properties [18,23,24,32,33].

The inhibition of pathogen adhesion to the host epithelium is a mechanism of great interest in the prevention of various diseases, as it represents a critical and essential step in bacterial pathogenesis [34]. Therefore, adhesion to the epithelium is an important selection criterion, as it provides a competitive advantage for binding sites on the intestinal mucosa. In this context, a family of cell wall glycoproteins known as “adhesins” has been identified in many fungi, endowing them with unique adhesion properties. These proteins are crucial for interactions between fungal cells and for communication with other cells, including those of the host [35].

Certain properties related to in vitro adhesion, such as hydrophobicity, biofilm formation and auto aggregation, were previously evaluated in wine yeasts, yielding low, moderate, and high values respectively in most cases [18]. *K. marxianus* CIDCA 8154 and CIDCA 9121 were also hydrophilic when grown in whey, with a hydrophobicity percentage lower than 10 (unpublished results).

In general, high hydrophobicity is commonly considered to correlate with good adhesion properties. However, our study did not align with this expectation, as yeast strains exhibited good adhesion, with values ranging from 33–100% adherence to Caco-2/TC-7 cells despite their low hydrophobicity. In contrast to our findings, several authors reported low to moderate adherence (<14%) for different *Kluyveromyces* and *Pichia* strains [36,37,38,39,40,41,42].

Consistent with our reports, other authors have documented adherence percentages exceeding 50% in different *K. marxianus* strains [43,44]. It is still unclear whether this capacity should be considered a mandatory criterion for probiotic status, as there are studies demonstrating that probiotic mechanisms of action can be exerted without strong adherence. In cases of low adherence, continuous oral administration is necessary to exert probiotic effects, as the cessation of probiotic use leads to their gradual disappearance from the intestine.

Although significant variability was observed in the adhesion capacity of the six evaluated strains, the findings in this study are very promising. Interestingly, no results have been previously shown regarding the adhesion of the *Wickerhamomyces* genus to epithelial cells, being this the first report. The results demonstrate that in vitro adhesion capacity is a strain-specific characteristic. It is important to note that each yeast cell can possess several different adhesins, and the differential expression of their associated genes is highly adapted to a particular environment [22], which could explain the observed differences.

The association and invasion of *Salmonella* spp. to the intestinal epithelium both represent a critical step in initiating the infection. In the present work, the antimicrobial activity of kefir and wine yeast strains was evaluated under three treatments to protect the intestinal epithelium against damage caused by *S. Enteritidis*: yeast effect (YE), *Salmonella* pretreatment with yeast culture (CC), and *Salmonella* pretreatment with cell-free culture supernatant (CFS). In general, the assays demonstrated that when yeasts and *S. Enteritidis* were co-cultured, the adhesion and invasion capacity of this pathogen to Caco-2/TC-7 cells decreases, showing the antagonistic effect of yeasts on bacteria in the invasion of intestinal cells. However, the three treatments were very effective in inhibiting the invasion of *Salmonella* to Caco-2/TC-7 cells, since a significant reduction was evidenced in most conditions. Despite the fact that the competition for binding sites in the intestinal mucosa appears to be a common protective effect of probiotics against enteropathogens, the results obtained in this study demonstrate that the antagonism against *Salmonella,* as evidenced by these yeast strains, is primarily mediated by the complex interaction between probiotics and the pathogen rather than a simple barrier effect. These interactions may involve both cell contact and the production of metabolites by the yeast; this could impact the surface adhesins and invasins expressed by *Salmonella,* thus reducing the risk of infection. In this context, there is evidence supporting the idea that *Saccharomyces boulardii* exerts biocontrol against *Clostridium difficile* through proteolytic activity and steric hindrance [45]. On the other hand, Fu et al. suggest that the yeasts’ antibacterial activity may originate from protein metabolites, such as certain enzymes, killer toxins, or mycocins, as well as organic acids like acetic acid [46].

Intestinal epithelial cells and tight junctions constitute the first line of defense against the invasion of pathogenic microorganisms and toxic compounds. Their alteration implies entry into the intestinal lumen (paracellular permeability) and, ultimately, circulatory and tissue invasion [47]. These alterations lead to the activation of inflammatory pathways and elevated levels of proinflammatory cytokines [48].

Chemotaxis is a known virulence factor in a wide range of pathogens, enabling bacteria to optimize nutrient acquisition, avoid toxic substances, and/or move to ideal infection sites [49]. The data suggest that *Salmonella* sp. uses chemotaxis to advance towards a more ideal metabolic niche in the intestine, conferring competitive advantages [50,51]. *Salmonella* sp. intentionally induces inflammation by injecting proteins through the T3SS. These include specific effectors leading to the induction of the NfκB pathway [52], as well as immune detection of both flagellin and T3SS per se [53,54,55]. Inflammation results in increased mucin production, containing high-energy glycoconjugates and amino acids that can be used as carbon sources for *Salmonella* sp. [56]. Additionally, there is an influx of neutrophils into the intestinal lumen that produce reactive oxygen species (ROS), reacting with available thiosulfate to generate tetrathionate (S_4_O_6_**^−^**^2^), a terminal electron acceptor only usable by a limited group of bacteria, including *Salmonella* sp. [57]. Therefore, *Salmonella* sp. benefits from the inflammatory response by utilizing recently available carbon sources and electron acceptors. Furthermore, during intestinal inflammation, *Salmonella* sp. can respire using tetrathionate and nitrate (a byproduct of the inflammatory response), thus gaining access to more carbon sources, including non-fermentable ethanolamine and propanediol (available in the intestine), and competitively overcoming fermentative bacteria residing in the host’s intestinal microbiota [58,59,60,61].

Immunomodulation is one of the modes of action employed by probiotic yeasts to control pathogens. It has been confirmed that β-glucans, natural glucose polymers present in the cell walls of certain yeasts, have stimulating effects on innate immune cells such as macrophages, neutrophils, and natural killer cells, as well as antibacterial and antitumor activities, and the induction of cytokine production [62]. Other studies have indicated that some yeasts can modulate the immune system to reduce inflammation and counteract enteropathogenesis [16,24]. The chemokine CCL20 is expressed in the gastrointestinal tract under proinflammatory conditions such as infections and inflammatory bowel disease [63]. In our screening system, all yeast strains analyzed were capable of inhibiting flagellin-induced activation, with strains from the *Kluyveromyces* and *Pichia* genera being the most immunomodulatory. Previous reports have indicated that kefir yeasts have a high capacity to inhibit the innate response of the intestinal epithelium triggered by different proinflammatory stimuli in vitro [23]. It is noteworthy that growth in whey permeate does not modify this biological activity of *K. marxianus* CIDCA 8154 and CIDCA 9121 [12]. To date, no studies have been reported on oenological strains of the studied genus with immunomodulatory properties. It is believed that the health-promoting properties of probiotic microorganisms are strain-specific. In fact, members of the same species can induce proinflammatory or anti-inflammatory activity, differentially modulate the intestinal microbiota, or induce specific antimicrobial effects [64,65]. As mentioned earlier, one of the pathogenesis mechanisms of *Salmonella* sp. is the induction of inflammation, so the results obtained in this study are highly relevant, providing evidence that the six analyzed strains from different fermentative environments and belonging to different genera have the ability to modulate the innate inflammatory immune response, which is an antagonistic mechanism against this enteropathogen.

## 5. Conclusions

This study confirms that fermented beverages could be a source of yeasts with probiotic potential. The six assayed strains exhibited antagonistic activity against *Salmonella* Enteritidis through different modes of action, indicating that they would not only exert a common mechanism but also there could be a complex and specific interaction between each of them and the pathogenic microorganism. Moreover, all strains behaved differently in the diverse models of interaction studied, highlighting strain-dependent behaviors. In particular for oenological *W. anomalus* and *P. manshurica*, this is the first time such studies have been reported, providing valuable evidence about their significant probiotic potential.

The results of the present work are relevant for the development of new alternatives to antibiotics to counteract the action of this pathogen. However, in the near future, in vivo experiments need to be performed in order to validate these results.

## Figures and Tables

**Table 1:** Origin, nomenclature, percentage of adhesion of yeasts to Caco-2/TC-7 cells, and auto-aggregation coefficient of yeast after 1 h.

Origin	Genus and Specie	Strain	Adhesion to Caco-2/TC-7 Cells (%)	Auto-Aggregation Coefficient
Grape musts	*Wickerhamomyces anomalus*	PB 98	56.10 ± 6.90 ^a,c^	1.59 ± 0.91
Grape musts	*W. anomalus*	PB 99	75.93 ± 2.61 ^a,b^	2.04 ± 0.17
Grape musts	*Pichia manshurica*	PB 54	47.56 ± 9.69 ^a,c^	4.34 ± 1.46
Grape musts	*W. anomalus*	PB 97	100.00 ± 6.43 ^b^	4.01 ± 1.72
Kefir grains	*Kluyveromyces marxianus*	CIDCA 8154	33.89 ± 5.50 ^c^	91.99 ± 6.01
Sugary kefir grains	*K. marxianus*	CIDCA 9121	73.33 ± 9.43 ^a,b^	88.10 ± 6.40

Different letters indicate significance differences (*p* < 0.05, Tukey’s multiple comparison test).

## Data Availability

The raw data supporting the conclusions of this article will be made available by the authors on request.

## Supplementary Materials

The following supporting information can be downloaded at: https://www.mdpi.com/article/10.3390/jof10120878/s1, Figure S1: Auto-aggregation test. The OD at 610 nm of a suspension of 10^7^ CFU/mL of each yeast strain was measured every 10 min for 60 min without disturbances. Figure S2: Co-aggregation test. The OD at 610 nm of a mixed suspension of 10^7^ CFU/mL of each yeast strain and 10^7^ CFU/mL of *S. Enteritidis* was measured every 10 min for 60 min without disturbances (black square). Auto-aggregation test is also shown for each yeast (black circle) and for *S. Enteritidis* (black triangle).

## References

[1] Wang G., Song Q., Huang S., Wang Y., Cai S., Yu H., Ding X., Zeng X., Zhang J. (2020). Effect of antimicrobial peptide microcin J25 on growth performance, immune regulation, and intestinal microbiota in broiler chickens challenged with *Escherichia coli* and *Salmonella*. Animals.

[2] Bonnet V., Dupont H., Glorion S., Aupée M., Kipnis E., Gérard J.L., Hanouz J.L., Fischer M.O. (2019). Influence of bacterial resistance on mortality in intensive care units: A registry study from 2000 to 2013 (IICU Study). J. Hosp. Infect..

[3] Gut A.M., Vasiljevic T., Yeager T., Donkor O.N. (2018). *Salmonella* infection—Prevention and treatment by antibiotics and probiotic yeasts: A review. Microbiology.

[4] Mullis M.M., Rambo I.M., Baker B.J., Reese B.K. (2019). Diversity, ecology, and prevalence of antimicrobials in nature. Front. Microbiol..

[5] Walsham A. (2016). Determining the Protective Effects of *Lactobacillus reuteri* Against Enteropathogenic *Escherichia coli* Infection. Ph.D. Thesis.

[6] Tuo Y., Song X., Song Y., Liu W., Tang Y., Gao Y., Mu G. (2018). Screening probiotics from *Lactobacillus* strains according to their abilities to inhibit pathogen adhesion and induction of pro-inflammatory cytokine IL-8. J. Dairy Sci..

[7] Wiese J., Imhoff J.F. (2019). Marine bacteria and fungi as promising source for new antibiotics. Drug Dev. Res..

[8] Hill C., Guarner F., Reid G., Gibson G.R., Merenstein D.J., Pot B., Morelli L., Canani R.B., Flint H.J., Salminen S. (2014). The International Scientific Association for Probiotics and Prebiotics consensus statement on the scope and appropriate use of the term probiotic. Nat. Rev. Gastroenterol. Hepatol..

[9] Helmy Q., Kardena E., Gustiani S., Blumenberg M., Shaaban M., Elgalml A. (2019). Probiotics and bioremediation. Microorganisms.

[10] Ayyash M.M., Abdalla A.K., AlKalbani N.S., Baig M.A., Turner M.S., Liu S.Q., Shah N.P. (2021). Invited review: Characterization of new probiotics from dairy and nondairy products—Insights into acid tolerance, bile metabolism and tolerance, and adhesion capability. J. Dairy. Sci..

[11] Singh R.P., Shadan A., Ma Y. (2022). Biotechnological applications of probiotics: A multifarious weapon to disease and metabolic abnormality. Probiotics Antimicrob. Proteins.

[12] Pendón M.D., Madeira J.V., Romanin D.E., Rumbo M., Gombert A.K., Garrote G.L. (2021). A biorefinery concept for the production of fuel ethanol, probiotic yeast, and whey protein from a by-product of the cheese industry. Appl. Microbiol. Biotechnol..

[13] Staniszewski A., Kordowska-Wiater M. (2021). Probiotic and potentially probiotic yeasts—Characteristics and food application. Foods.

[14] Sen S., Mansell T.J. (2020). Yeasts as probiotics: Mechanisms, outcomes, and future potential. Fungal Genet. Biol..

[15] Ianiro G., Bruno G., Lopetuso L., Beghella Bartoli F., Laterza L., D’Aversa F., Gigante G., Cammarota G., Gasbarrini A. (2014). Role of yeasts in healthy and impaired gut microbiota: The gut mycome. Curr. Pharm. Des..

[16] González-Orozco B.D., García-Cano I., Jiménez-Flores R., Alvárez V.B. (2022). Invited review: Milk kefir microbiota—Direct and indirect antimicrobial effects. J. Dairy Sci..

[17] Collado M.C., Lee Y.K., Salminen S. (2019). Role of probiotics in health and diseases. Handbook of Probiotics and Prebiotics.

[18] Vergara S.C., Leiva Alaniz M.J., Mestre Furlani M.V., Vazquez F., Mancha Agresti P., Nally M.C., Maturano Y.P. (2023). Bioprospecting of the probiotic potential of yeasts isolated from a wine environment. Fungal Genet. Biol..

[19] Kos B.V.Z.E., Šušković J., Vuković S., Šimpraga M., Frece J., Matošić S. (2003). Adhesion and aggregation ability of probiotic strain *Lactobacillus acidophilus* M92. J. Appl. Microbiol..

[20] Papadimitriou K., Zoumpopoulou G., Foligné B., Alexandraki V., Kazou M., Pot B., Tsakalidou E. (2015). Discovering probiotic microorganisms: In vitro, in vivo, genetic and omics approaches. Front. Microbiol..

[21] Alkalbani N.S., Osaili T.M., Al-Nabulsi A.A., Olaimat A.N., Liu S.-Q., Shah N.P., Apostolopoulos V., Ayyash M.M. (2022). Assessment of yeasts as potential probiotics: A review of gastrointestinal tract conditions and investigation methods. J. Fungi.

[22] Verstrepen K.J., Klis F.M. (2006). Flocculation, adhesion and biofilm formation in yeasts. Mol. Microbiol..

[23] Romanin D., Serradell M., González Maciel D., Lausada N., Garrote G.L., Rumbo M. (2010). Down-regulation of intestinal epithelial innate response by probiotic yeasts isolated from kefir. Int. J. Food Microbiol..

[24] Diosma G., Romanin D.E., Rey-Burusco M.F., Londero A., Garrote G.L. (2014). Yeasts from kefir grains: Isolation, identification, and probiotic characterization. World J. Microbiol. Biotechnol..

[25] Vergara S.C., Leiva M.J., Mestre M.V., Vazquez F., Nally M.C., Maturano Y.P. (2023). Non-*Saccharomyces* yeast probiotics: Revealing relevance and potential. FEMS Yeast Res..

[26] Kurtzman C., Fell J.W., Boekhout T. (2011). The Yeasts: A Taxonomic Study.

[27] Esteve-Zarzoso B., Belloch C., Uruburul F., Querol A. (1999). Identification of yeasts by RFLP analysis of the 5.85 rRNA gene and the two ribosomal internal transcribed spacers. Int. J. Syst. Bacteriol..

[28] Zavala L., Golowczyc M.A., Van Hoorde K., Medrano M., Huys G., Vandamme P., Abraham A.G. (2016). Selected *Lactobacillus* strains isolated from sugary and milk kefir reduce *Salmonella* infection of epithelial cells in vitro. Benef. Microbes.

[29] Prabhurajeshwar C., Chandrakanth R.K. (2017). Probiotic potential of Lactobacilli with antagonistic activity against pathogenic strains: An in vitro validation for the production of inhibitory substances. Biomed. J..

[30] Nempont C., Cayet D., Rumbo M., Bompard C., Villeret V., Sirard J.-C. (2008). Deletion of flagellin’s hypervariable region abrogates antibody-mediated neutralization and systemic activation of TLR5-dependent immunity. J. Immunol..

[31] Iraporda C., Romanin D.E., Rumbo M., Garrote G.L., Abraham A.G. (2014). The role of lactate on the immunomodulatory properties of the nonbacterial fraction of kefir. Food Res. Int..

[32] Garrote G.L., Abraham A.G., De Antoni G.L. (2001). Chemical and microbiological characterisation of kefir grains. J. Dairy Res..

[33] Romanin D.E., Llopis S., Genovés S., Martorell P., Ramón V.D., Garrote G.L., Rumbo M. (2016). Probiotic yeast *Kluyveromyces marxianus* CIDCA 8154 shows anti-inflammatory and anti-oxidative stress properties in in vivo models. Benef. Microbes.

[34] Xu H., Jeong H.S., Lee H.Y., Ahn J. (2009). Assessment of cell surface properties and adhesion potential of selected probiotic strains. Lett. Appl. Microbiol..

[35] Willaert R.G. (2018). Adhesins of yeasts: Protein structure and interactions. J. Fungi.

[36] Kumura H., Tanoue Y., Tsukahara M., Tanaka T., Shimazaki K. (2004). Screening of dairy yeast strains for probiotic applications. J. Dairy Sci..

[37] Chen L.S., Ma Y., Maubois J.L., He S.H., Chen L.J., Li H.M. (2010). Screening for the potential probiotic yeast strains from raw milk to assimilate cholesterol. Dairy Sci. Technol..

[38] Fadda M.E., Mossa V., Deplano M., Pisano M.B., Cosentino S. (2017). In vitro screening of *Kluyveromyces* strains isolated from Fiore Sardo cheese for potential use as probiotics. LWT.

[39] Bonatsou S., Karamouza M., Zoumpopoulou G., Mavrogonatou E., Kletsas D., Papadimitriou K., Tsakalidou E., Nychas G.J.E., Panagou E. (2018). Evaluating the probiotic potential and technological characteristics of yeasts implicated in cv. Kalamata natural black olive fermentation. Int. J. Food Microbiol..

[40] Menezes A.G.T., Ramos C.L., Cenzi G., Melo D.S., Dias D.R., Schwan R.F. (2020). Probiotic potential, antioxidant activity, and phytase production of indigenous yeasts isolated from indigenous fermented foods. Probiotics Antimicrob. Proteins.

[41] Motey G.A., Johansen P.G., Owusu-Kwarteng J., Ofori L.A., Obiri-Danso K., Siegumfeldt H., Larsen N., Jespersen L. (2020). Probiotic potential of *Saccharomyces cerevisiae* and *Kluyveromyces marxianus* isolated from West African spontaneously fermented cereal and milk products. Yeast.

[42] González-Orozco B.D., Kosmerl E., Jiménez-Flores R., Alvarez V.B. (2023). Enhanced probiotic potential of *Lactobacillus kefiranofaciens* OSU-BDGOA1 through co-culture with *Kluyveromyces marxianus* bdgo-ym6. Front. Microbiol..

[43] Maccaferri S., Klinder A., Brigidi P., Cavina P., Costabile A. (2012). Potential probiotic *Kluyveromyces marxianus* B0399 modulates the immune response in Caco-2 cells and peripheral blood mononuclear cells and impacts the human gut microbiota in an in vitro colonic model system. Appl. Environ. Microbiol..

[44] Simões L.A., Cristina de Souza A., Ferreira I., Melo D.S., Lopes L.A.A., Magnani M., Schwan R.F., Dias D.R. (2021). Probiotic properties of yeasts isolated from Brazilian fermented table olives. J. Appl. Microbiol..

[45] Tasteyre A., Barc M.C., Karjalainen T., Bourlioux P., Collignon A. (2002). Inhibition of in vitro cell adherence of *Clostridium difficile* by *Saccharomyces boulardii*. Microb. Pathog..

[46] Fu J.J., Liu J., Wen X.P., Zhang G., Cai J., Qiao Z., An Z., Zheng J., Li L. (2023). Unique probiotic properties and bioactive metabolites of *Saccharomyces boulardii*. Probiotics Antimicrob. Proteins.

[47] Slifer Z.M., Blikslager A.T. (2020). The integral role of tight junction proteins in the repair of injured intestinal epithelium. Int. J. Mol. Sci..

[48] Bhat A.A., Uppada S., Achkar I.W., Hashem S., Yadav S.K., Shanmugakonar M., Al-Naemi H.A., Haris M., Uddin S. (2019). Tight junction proteins and signaling pathways in cancer and inflammation: A functional crosstalk. Front. Physiol..

[49] Ottemann K.M., Miller J.F. (1997). Roles for motility in bacterial–host interactions. Mol. Microbiol..

[50] Rivera-Chávez F., Winter S.E., Lopez C.A., Xavier M.N., Winter M.G., Nuccio S.P., Russell J.M., Laughlin R.C., Lawhon S.D., Sterzenbach T. (2013). *Salmonella* uses energy taxis to benefit from intestinal inflammation. PLoS Pathog..

[51] Palmer A.D., Slauch J.M. (2017). Mechanisms of *Salmonella* pathogenesis in animal models. Hum. Ecol. Risk Assess. Int. J..

[52] Keestra M.A., Winter M.G., Klein-Douwel D., Xavier M.N., Winter S.E., Kim A., Tsolis R.M., Bäumler A.J. (2011). A *Salmonella* virulence factor activates the NOD1/NOD2 signaling pathway. mBio.

[53] Crowley S.M., Knodler L.A., Vallance B.A., Backert S. (2016). Salmonella and the inflammasome: Battle for intracellular dominance. Inflammasome Signaling and Bacterial Infections.

[54] Sellin M.E., Maslowski K.M., Maloy K.J., Hardt W.D. (2015). Inflammasomes of the intestinal epithelium. Trends Immunol..

[55] Winter S.E., Thiennimitr P., Nuccio S.P., Haneda T., Winter M.G., Wilson R.P., Russell J.M., Henry T., Quynh T.T., Lawhon S.D. (2009). Contribution of flagellin pattern recognition to intestinal inflammation during *Salmonella* enterica serotype typhimurium infection. Infect. Immun..

[56] Stecher B., Hardt W.D. (2008). The role of microbiota in infectious disease. Trends Microbiol..

[57] Winter S.E., Thiennimitr P., Winter M.G., Butler B.P., Huseby D.L., Crawford R.W., Russell M.R., Bevins C.L., Adams L.G., Tsolis R.M. (2010). Gut inflammation provides a respiratory electron acceptor for *Salmonella*. Nature.

[58] Thiennimitr P., Winter S.E., Winter M.G., Xavier M.N., Tolstikov V., Huseby D.L., Sterzenbach T., Tsolis R.M., Roth J.R., Bäumler A.J. (2011). Intestinal inflammation allows *Salmonella* to use ethanolamine to compete with the microbiota. Proc. Natl. Acad. Sci. USA.

[59] Lopez C.A., Winter S.E., Rivera-Chávez F., Xavier M.N., Poon V., Nuccio S.P., Tsolis R.M., Bäumler A.J. (2012). Phage-mediated acquisition of a type III secreted effector protein boosts growth of *Salmonella* by nitrate respiration. mBio.

[60] Winter S.E., Winter M.G., Xavier M.N., Thiennimitr P., Poon V., Keestra A.M., Laughlin R.C., Gomez G., Wu J., Lawhon S.D. (2013). Host-derived nitrate boosts growth of *E. coli* in the inflamed gut. Science.

[61] Faber F., Thiennimitr P., Spiga L., Byndloss M.X., Litvak Y., Lawhon S., Andrews-Polymenis H.L., Winter S.E., Bäumler A.J. (2017). Respiration of microbiota-derived 1, 2-propanediol drives *Salmonella* expansion during colitis. PloS Pathog..

[62] Shruthi B., Deepa N., Somashekaraiah R., Adithi G., Divyashree S., Sreenivasa M.Y. (2022). Exploring biotechnological and functional characteristics of probiotic yeasts: A review. Biotechnol. Rep..

[63] Rumbo M., Sierro F., Debard N., Kraehenbuhl J.P., Finke D. (2004). Lymphotoxin β receptor signaling induces the chemokine CCL20 in intestinal epithelium. Gastroenterology.

[64] Ramos J.M.O., Santos C.A., Santana D.G., Santos D.A., Alves P.B., Thomazzi S.M. (2013). Chemical constituents and potential anti-inflammatory activity of the essential oil from the leaves of Croton argyrophyllus. Rev. Bras. Farmacogn..

[65] Su X., Zhang J., Wang H., Xu J., He J., Liu L., Zhang T., Chen R., Kang J. (2017). Phenolic acid profiling, antioxidant, and anti-inflammatory activities, and miRNA regulation in the polyphenols of 16 blueberry samples from China. Molecules.

