# Supplementary material for: Probiotic Potential of Yeasts Isolated from Fermented Beverages: Assessment of Antagonistic Strategies Against Salmonella enterica Serovar Enteritidis
Source: J Fungi (Basel). 2024 Dec 17;10(12):878. doi: 10.3390/jof10120878 (PMC11676645; doi:10.3390/jof10120878)
Supplement: Supplementary file 1 [file jof-10-00878-s001.zip › jof-3331339-supplementary.pdf]

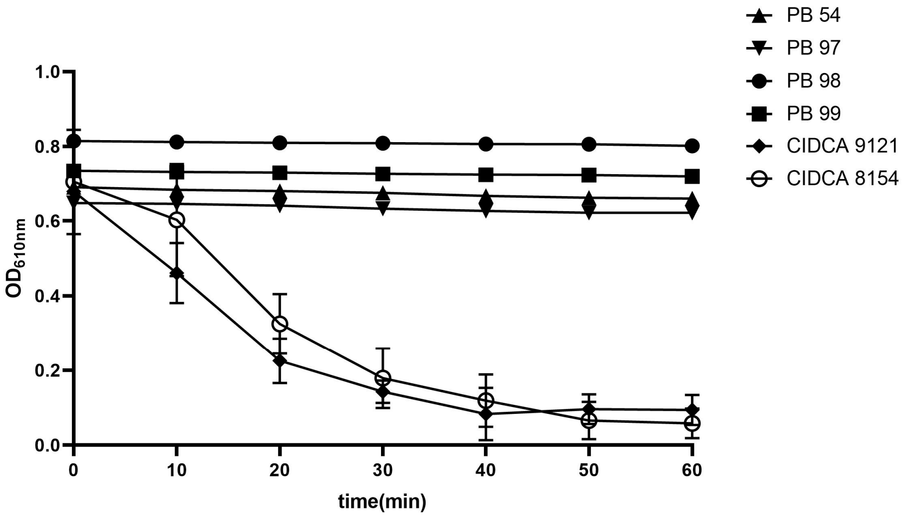

Figure S1: Auto-aggregation test. The OD at 610 nm of a suspension of 10^7 CFU/mL of each yeast strain was measured every 10 min for 60 min without disturbances.

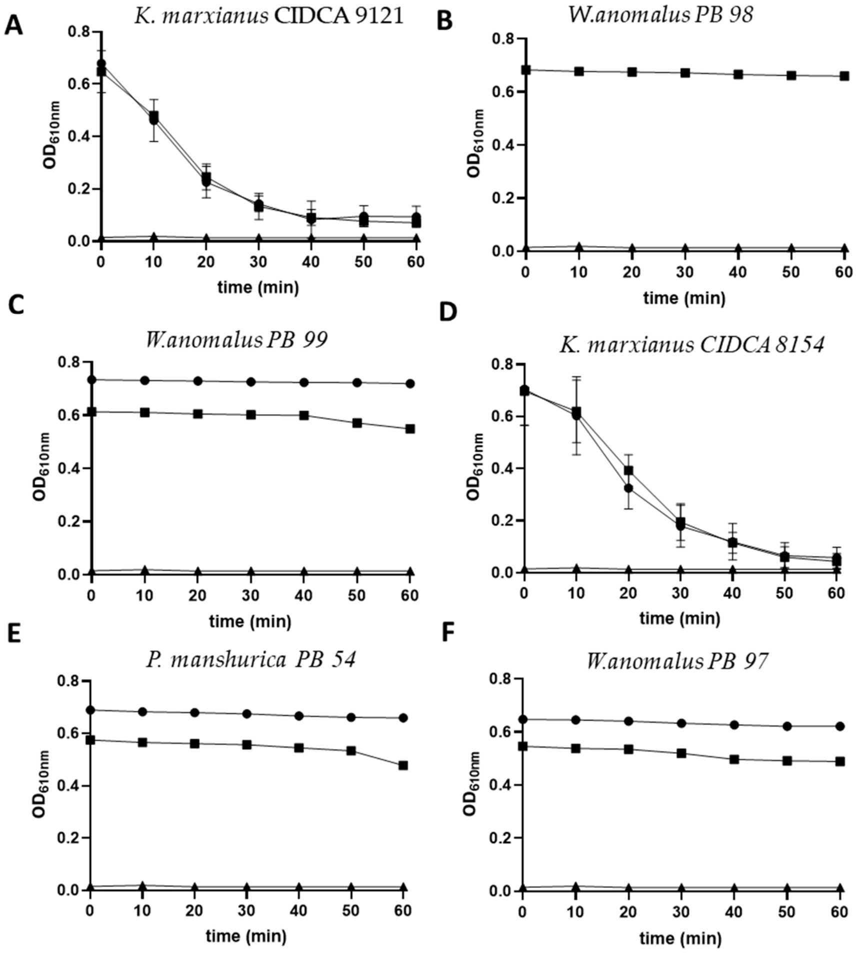

Figure S2: Co-aggregation test. The OD at 610 nm of a mixed suspension of 10^7 CFU/mL of each yeast strain and 10^7 CFU/mL of *S.* Enteritidis was measured every 10 min for 60 min without disturbances (black square). Auto-aggregation test is also shown for each yeast (black circle) and for *S*. Enteritidis (black triangle)
